# Genomic Analysis of *Sarcomyxa edulis* Reveals the Basis of Its Medicinal Properties and Evolutionary Relationships

**DOI:** 10.3389/fmicb.2021.652324

**Published:** 2021-07-01

**Authors:** Fenghua Tian, Changtian Li, Yu Li

**Affiliations:** ^1^Department of Plant Pathology, College of Agriculture, Guizhou University, Guiyang, China; ^2^Engineering Research Center of Chinese Ministry of Education for Edible and Medicinal Fungi, Jilin Agricultural University, Changchun, China

**Keywords:** edible-medicinal mushroom, lignin-degrading peroxidases, phylogenetic analyses, *Sarcomyxa edulis*, secondary metabolism

## Abstract

Yuanmo [*Sarcomyxa edulis* (Y.C. Dai, Niemelä & G.F. Qin) T. Saito, Tonouchi & T. Harada] is an important edible and medicinal mushroom endemic to Northeastern China. Here we report the *de novo* sequencing and assembly of the *S. edulis* genome using single-molecule real-time sequencing technology. The whole genome was approximately 35.65 Mb, with a G + C content of 48.31%. Genome assembly generated 41 contigs with an N50 length of 1,772,559 bp. The genome comprised 9,364 annotated protein-coding genes, many of which encoded enzymes involved in the modification, biosynthesis, and degradation of glycoconjugates and carbohydrates or enzymes predicted to be involved in the biosynthesis of secondary metabolites such as terpene, type I polyketide, siderophore, and fatty acids, which are responsible for the pharmacodynamic activities of *S. edulis*. We also identified genes encoding 1,3-β-glucan synthase and endo-1,3(4)-β-glucanase, which are involved in polysaccharide and uridine diphosphate glucose biosynthesis. Phylogenetic and comparative analyses of Basidiomycota fungi based on a single-copy orthologous protein indicated that the *Sarcomyxa* genus is an independent group that evolved from the Pleurotaceae family. The annotated whole-genome sequence of *S. edulis* can serve as a reference for investigations of bioactive compounds with medicinal value and the development and commercial production of superior *S. edulis* varieties.

## Introduction

*Sarcomyxa edulis* (Y.C. Dai, Niemelä & G.F. Qin) T. Saito, Tonouchi & T. Harada is a fungus that is native to the temperate regions of Northeastern China, Northern Japan, United States, and Russian Far East (Jin et al., 2001; Dai et al., 2003; Saito et al., 2014). It is commonly known as the late oyster and is also called “Yuanmo,” “Huangmo,” or “Dongmo” in Chinese and “Mukitake” in Japanese. The fruiting body of *S. edulis* is fan-shaped, which is similar to *Ganoderma lucidum* (“Lingzhi”), but because of its yellow color, it is also known as “Huanglinggu” in China.

*Sarcomyxa edulis* is an edible and medicinal fungus (Imazeki et al., 1988; Li, 1991; Pan, 1995) that is prized for its nutritional value, unique aroma, delicate flavor, and meaty texture as well as its medicinal properties (Li, 1991; Ma et al., 1991; Pan, 1995; Inafuku et al., 2012; Kim et al., 2012; Inoue et al., 2013; Li et al., 2013, 2016; Zhang et al., 2014). In traditional Chinese medicine, *S. edulis* is prepared as an alcohol decoction to treat stomachache and other ailments.

*Sarcomyxa edulis* was first described and assigned to the genus *Panellus* (Dai et al., 2003) through a comparison with the terribly bitter mushrooms from Finland to the specimens of China. *S. edulis* is under the same genus as *Sarcomyxa serotina*. As the type species, the taxonomic status of *S. serotina* has changed multiple times from the *Agaricus* genus to *Pleurotus*, *Sarcomyxa*, *Hohenbuehelia*, and finally *Panellus* (Dai et al., 2003). Phylogenetic studies have shown that *S. serotina* and *S. edulis* are excluded from the *Panellus* clade. A maximum likelihood (ML) tree constructed based on the D1/D2 region of the large subunit of the 28S ribosomal RNA gene showed that the *Sarcomyxa* genus formed a clade that was independent of *Mycena* and *Panellus* (Matheny et al., 2006; Saito et al., 2014). Thus, the classification of *Sarcomyxa* is controversial.

*Sarcomyxa edulis* was domesticated in the last decade in China; like *Hericium erinaceus*, it is an important specialty mushroom cultivated in Northern China, the largest production region. The commercial cultivation of *S. edulis* is profitable because of high demand. However, production has markedly declined in recent years as a result of diseases and the lack of resistant varieties.

Knowledge of the biology and evolution of *S. edulis* is limited because molecular-level information from the genome is lacking. The availability of a whole-genome sequence can clarify the taxonomy of *S. edulis* and aid future breeding efforts to improve this species for commercial cultivation. To this end, in the present study, we sequenced and annotated the genome of a monokaryotic strain of *S. edulis* and carried out a phylogenetic analysis that included 32 sequenced fungi, with the aim of establishing the taxonomic status of *S. edulis* and identifying secondary metabolites of medicinal importance.

## Materials and Methods

### *Sarcomyxa edulis* Specimens

**FIGURE 1:**
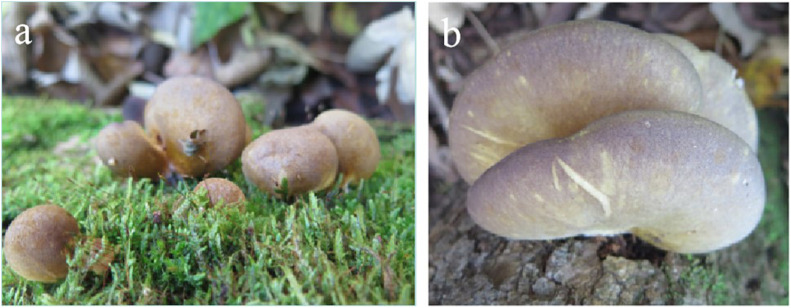
Fruiting body of *Sarcomyxa edulis*. **(a)** Developmental stages of *S. edulis*. **(b)** Mature *S. edulis*.

*Sarcomyxa edulis* basidiomes were collected from maple wood in Antu County, Jilin Province, China. Specimen 2016092521 was identified by morphologic and molecular analyses (Dai et al., 2003; Saito et al., 2014). The specimen was deposited in the herbarium of the Culture Center of Mycophyta in Jilin Agricultural University under accession number CCMJ18024 (Figure 1). The monokaryotic strain SE1 was germinated from one of the spores of specimen 2016092521 (Choi et al., 1999; Senanayake et al., 2020) and used for whole-genome sequencing. The mycelia of SE1 were cultured on an improved potato dextrose agar medium containing 5% wheat bran for 10 days at 24°C in the dark and collected for genome sequencing.

### Whole-Genome Sequencing

Total DNA of *S. edulis* strain SE1 was extracted using the NuClear Plant Genomic DNA kit (Tiangen Biotech, Beijing, China). The DNA was detected by agarose gel electrophoresis and quantified with a Qubit fluorometer (Thermo Fisher Scientific, Waltham, MA, United States). The whole genome of SE1 was sequenced using PacBio RSII Single Molecule Real-Time (SMRT) technology, which generated 20-kb SMRTbell libraries. High-throughput sequencing on an HiSeq PE150 system (Illumina, San Diego, CA, United States) was carried out to polish the DNA sequence; additionally, paired-end read libraries were obtained by sequencing 350-bp inserts.

Custom Novogene pipelines (Beijing, China) were used to filter the PacBio RSII reads; those of low complexity and quality were filtered out with SMRT portal v3.2.0, with default parameters (Berlin et al., 2015; Koren and Phillippy, 2015), and the resultant clean reads were *de novo* assembled into a continuous contig with no gaps. Pilon v1.22 was used to polish the assembled long reads with the clean Illumina short reads (Walker et al., 2014). Genome completeness was assessed using Benchmarking Universal Single-Copy Orthologs (v4.1.4; Simão et al., 2015). RepeatMasker version open-4.0.5 was used to detect and annotate dispersed repeat sequences (Saha et al., 2008). Tandem repeat (TR) sequences were analyzed with Tandem Repeat Finder v4.07b (Benson, 1999). rRNA sequences were predicted with rRNAmmer v1.2 (Lagesen et al., 2007), and tRNA genes and tRNA secondary structures were predicted with tRNAscan-SE v1.3 (Lowe and Eddy, 1997). Non-coding RNAs including small (s)RNAs, micro (mi)RNAs, and small nuclear (sn)RNAs were annotated with Rfam (Gardner et al., 2009; Nawrocki et al., 2009) using default parameters (Cui et al., 2016). *Ab initio* and homology-based gene prediction methods were used to annotate the repeat masked SE1 genome assembly. For homolog-based gene prediction, the protein sequences of *Agaricus bisporus* (Morin et al., 2012), *Coprinopsis cinerea* (Stajich et al., 2010), *Pleurotus ostreatus* (Qu et al., 2016), and *Schizophyllum commune* (Ohm et al., 2010) were downloaded from the National Center for Biotechnology Information (NCBI) database and aligned using tBLASTn. Subsequently, mapping results were merged, and gene structures were predicted using GeneWise (v. 2.2.0; Birney et al., 2004). *Ab initio* genes were predicted using Augustus (Stanke et al., 2006), Genescan (Burge and Karlin, 1997), GlimmerHMM (Majoros et al., 2004), and SNAP (Korf, 2004). The protein-coding genes predicted from the *ab initio* and homology-based gene prediction methods were integrated using GLEAN (Elsik et al., 2007).

### Gene Annotation and Functional Analysis

In order to determine the functions of the predicted genes, we compared homologous genes to protein and nucleotide sequences in the general functional BLAST databases. The basic steps of functional annotation were as follows: (1) the predicted gene protein sequences were compared with the functional databases by DIAMOND (with an *e*-value ≤ 10^–5^; Buchfink et al., 2014) and (2) filtering of alignment results: for the alignment results of each sequence, select the alignment result with the highest score (default identity ≥ 40%, coverage ≥ 40%) for annotation (Altschul et al., 1990).

Six gene and protein databases were used to predict gene functions including the NCBI Non-redundant Protein Database (nr; Li et al., 2002), Kyoto Encyclopedia of Genes and Genomes (KEGG; Kanehisa et al., 2004, 2006), Gene Ontology (GO; Ashburner et al., 2000), Eukaryotic Orthologous Groups (KOG; Tatusov et al., 2003), and Transporter Classification Database (TCDB; Saier et al., 2014).

Secondary metabolites were annotated using the antiSMASH (fungiSMASH option) database^1^ and NaPDoS^2^ (Ziemert et al., 2012; Weber et al., 2015; Blin et al., 2017). To validate the predicted results, the obtained gene clusters were manually verified. Each gene model within the database was searched with BLASTP and TBLASTN algorithms (Grigoriev et al., 2012). For polyketide synthase (PKS)/non-ribosomal peptide synthetase (NRPS) analysis, we selected a query type and entered the sequence data to identify ketosynthase KS and/or condensation C domains using the NaPDos pipeline^3^. Carbohydrate-active enzymes (CAZymes) were determined using the dbCAN 2 meta server (Zhang et al., 2018). Cytochrome P450 monooxygenase (P450) analysis was performed by searching a reference dataset^4^ using the BLASTP program (Nelson, 2009).

### Phylogenetic Tree Construction

In addition to *S. edulis*, 31 fungal species in the Basidiomycota and Ascomycota phyla were included in the phylogenetic analysis. Protein sequence data for four taxa were downloaded from the Joint Genome Institute genome database (Bao et al., 2013; Aronsen and Læssøe, 2016; Castanera et al., 2017), and sequences for the other 27 taxa were obtained from the NCBI database (Supplementary Table 1). BLASTP was used to compare the 32 species (Chen et al., 2016). OrthoMCL software (Li et al., 2003) and all-*versus*-all BLASTP were used with the parameters (*e*-value ≤ 1e-15, coverage ≥ 50%) to identify orthologous groups. Single-copy orthologs were extracted by Perl script (command line parameters of Gblocks: Gblocks proteins.fasta –b4 = 5 –b5 = h.), and sequence alignments were analyzed with MAFFT v7.158b (Katoh and Standley, 2013). ProTest was used to generate an optimal base substitution model with ML and neighbor-joining (NJ; Castresana, 2000; Miller et al., 2010). Several fossil calibration points fixed were used as references: the most recent common ancestor (MRCA) of *P. ostreatus*, *S. commune*, *Lentinula edodes*, *Panellus stipticus*, *Mycena albidolilacea*, *Termitomyces* sp., *Hypsizygus marmoreus*, *Volvariella volvacea*, *A. bisporus* var. *bisporus*, *A. bisporus* var. *burnettii*, *Leucoagaricus* sp., *C. cinerea*, *Laccaria bicolor*, *Hypholoma sublateritium*, *Hebeloma cylindrosporum*, *Gymnopilus dilepis*, and *Galerina marginata* diverged 123 million years ago (MYA), while the MRCAs of other taxonomic groups diverged at the following time points: *Serpula lacrymans* and *Coniophora olivacea*, 104 MYA; *Ganoderma sinens*, *Postia placenta*, *Wolfiporia cocos*, *Trametes versicolor*, and *Dichomitus squalens*, 122 MYA; *Tremella mesenterica* and *Cryptococcus neoformans*, 153 MYA; and *Ustilago maydis*, 273 MYA (Floudas et al., 2012). The final phylogenetic tree was constructed using RAxML (Stamatakis, 2014).

## Results

### Identification of Specimen 2016092521

Specimen 2016092521 was identified as *S. edulis*. It is mild-tasting with ventricose cystidia, while the related species *S. serotina* is very bitter-tasting with hymenial cystidia. The basidiospores of the specimen are slightly longer than in *S. serotina*, 4.5–6.0 μm × 1.0–1.3 μm (Supplementary Figure 1). Maximum parsimony phylogenetic tree based on ITS gene sequences showed that specimen 2016092521 clusters with *S. edulis* and is distinct to *S. serotina* (Supplementary Figure 2).

### Features of the *S. edulis* Genome

In total, 5,318 Mb of clean data were obtained, from which a 35.65-Mb assembly was obtained. The genome consisted of 41 contigs with N50 of 1,772,559 bp, N90 of 554,178 bp, and 48.31% G + C content (Table 1). A BLAST search of repeat sequences yielded 1,371,373 bp, covering 3.85% of the SE1 genome; meanwhile, interspersed nuclear elements and TRs accounted for 1.79 and 2.05% of the genome, respectively. Approximately 1.53% of the genome was long terminal repeats, 0.09% was DNA transposons, and 0.16% was long interspersed nuclear elements. The proportion of TRs in the assembled genome was 1.36%, while minisatellite and microsatellite DNA accounted for 0.60 and 0.01% of the genome, respectively.

**TABLE 1:** General features of the *Sarcomyxa edulis* genome.

Characteristics	
**Scaffold**	
Total number	41
Total length (bp)	35,651,721
N50 (bp)	1,772,559
N90 (bp)	554,178
Max length (bp)	4,140,795
Genome coverage	150
GC content (%)	48.31
**Genome**	
Gene total length (bp)	15,135,900
Gene number	9,364
Gene length/genome (%)	42.45
Average gene length (bp)	1,616

### Functional Annotation

There were 9,364 gene models predicted in the different databases with a total sequence length of 15,135,900 bp, accounting for 42.45% of the whole genome with an average sequence length of 1,616 bp. We predicted 143 tRNAs (13,216 bp), 14 rRNAs (29,757 bp), 37 snRNAs (3,044 bp), 143 miRNAs (8,449 bp), and six sRNAs (359 bp; Supplementary Table 2). Among the 143 tRNAs, 21 were putative pseudogenes and 122 were anticodon tRNAs corresponding to the 20 common amino acid codons.

**FIGURE 2:**
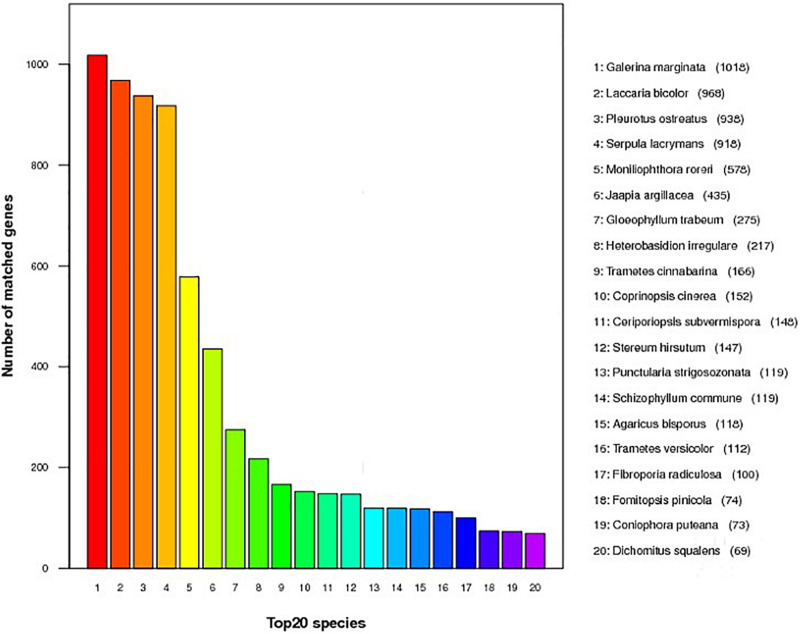
Predicted proteins from *Sarcomyxa edulis* genome to the National Center for Biotechnology Information non-redundant protein database among different fungal species.

Annotation was performed with the NCBI nr, KEGG, KOG, TCDB, GO, P450, Secretory Protein, and CAZy databases (Table 2 and Supplementary Table 3). In the nr database, 4,855 non-redundant proteins found in *S. edulis* had most matching with six fungi, accounting for 67.6% of total nr predicted proteins (Figure 2). These were similarly annotated in six species—namely, *G. marginata* (PRJNA207683), *L. bicolor* (PRJNA19043), *P. ostreatus* (PRJNA476433), *S. lacrymans* (PRJNA412961), *Moniliophthora roreri* (PRJNA279170), and *Jaapia argillacea* (PRJNA207685).

**TABLE 2:** Summary of *Sarcomyxa edulis* gene annotations.

Database used for gene/protein annotation	Number of genes
NR	7,183
GO	5,884
KEGG	2,954
KOG	1,760
Secretory protein	502
TCDB	290
CAZy	283
**Annotated gene**	
P450	162

*CAZy, Carbohydrate-active Enzymes database; GO, Gene Ontology; KEGG, Kyoto Encyclopedia of Genes and Genomes; KOG, Eukaryotic Orthologous Groups; P450, cytochrome P450 monooxygenase; nr, National Center for Biotechnology Information non-redundant protein database; and TCDB, Transporter Classification Database.*

**FIGURE 3:**
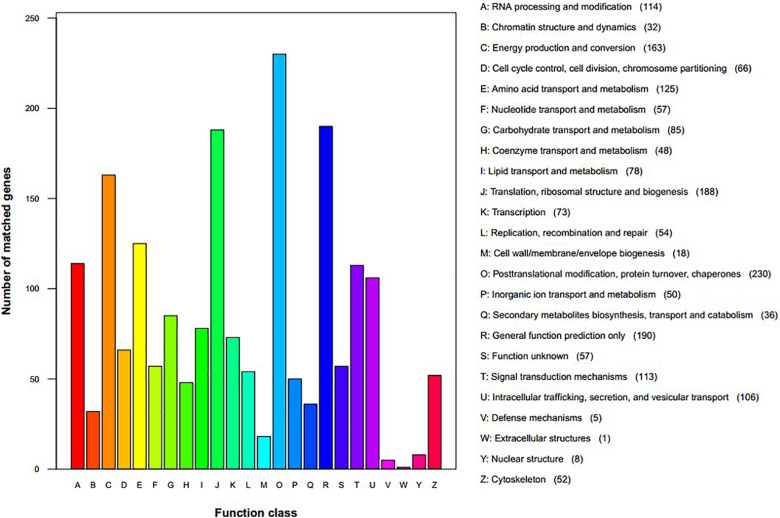
Eukaryotic Orthologous Groups functional classification of *Sarcomyxa edulis* proteins.

We assigned 1,760 proteins (18.80% of 9,364, the total annotated predicted proteins) to NCBI KOG categories (Figure 3). The “posttranslational modification, protein turnover, chaperones” category had the most enriched genes (230), followed by “general function prediction only” (190), “translation, ribosomal structure and biogenesis” (188), “energy production and conversion” (163), “amino acid transport and metabolism” (125), “RNA processing and modification” (124), “signal transduction mechanisms” (113), and “intracellular trafficking, secretion, and vesicular transport” (106). The representation of genes related to protein and energy metabolism could reflect the capacity of *S. edulis* to absorb and transform nutrients from a variety of substrates.

**FIGURE 4:**
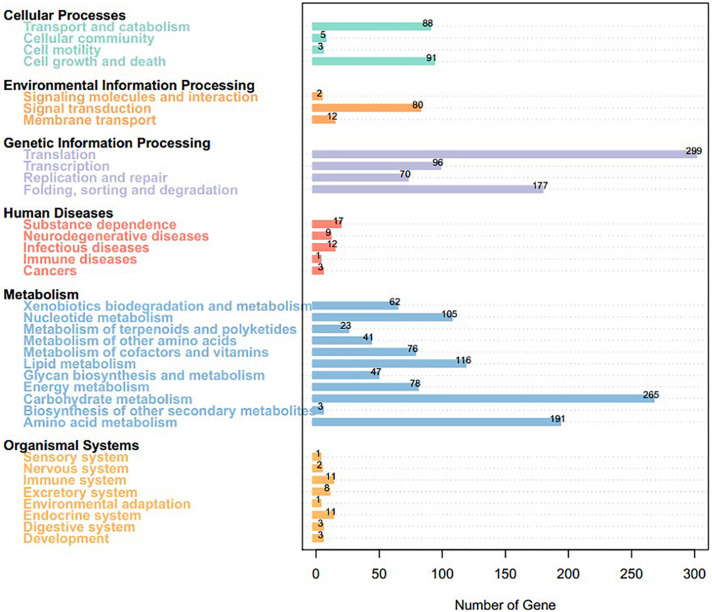
Kyoto Encyclopedia of Genes and Genomes pathway annotation of *Sarcomyxa edulis* genes.

We mapped the predicted genes to the KEGG database and assigned functional classifications to 2,954 (31.55%, 9,364 gene models; Figure 4). Some categories related to metabolism and biosynthesis were highly enriched, including “purine metabolism,” “oxidative phosphorylation,” “butanoate metabolism,” “fructose and mannose metabolism,” “starch and sucrose metabolism,” “amino sugar and nucleotide sugar metabolism,” “arginine and proline metabolism,” “pyrimidine metabolism,” and “chloroalkane and chloroalkene degradation.”

**FIGURE 5:**
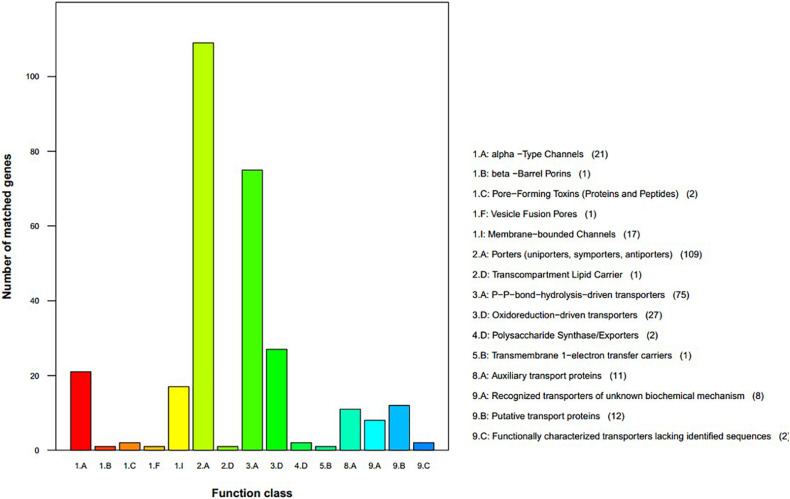
Transporter Classification Database functional annotation of *Sarcomyxa edulis* genes.

We used the TCDB to perform protein domain analysis and assigned 290 putative transport proteins to seven functional classes including “accessory factors involved in transport,” “channels/pores,” “electrochemical potential-driven transporters,” “group translocators,” “incompletely characterized transport systems,” “primary active transporters,” and “transmembrane electron carriers” (Figure 5). The top two enriched categories were “porters (uniporters, symporters, antiporters)” and “P–P bond hydrolysis-driven transporters.”

**FIGURE 6:**
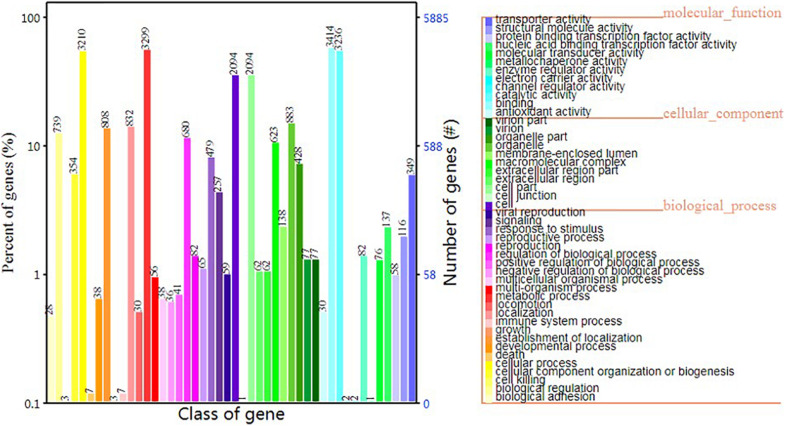
Gene Ontology functional annotation of *Sarcomyxa edulis* genes.

In terms of GO functional classes, we predicted 5,884 proteins that accounted for 62.84% of the total predicted proteins. The most highly enriched GO terms in *S. edulis* were “cell,” “cell part,” “cellular process,” “catalytic activity,” “metabolic process,” and “binding” (Figure 6).

Cytochrome P450 (CYP) is a superfamily of hemoproteins that use heme as a cofactor. CYPs have various substrates in different enzymatic reactions and are present in all kingdoms. We identified 83 putative CYP genes in *S. edulis* through a BLAST search and classified them into 34 families (Supplementary Table 4). The CYP5144 family had the highest number of enriched genes (14), followed by CYP5037 (13).

The CAZymes play important roles in the degradation of renewable lignocelluloses to provide carbohydrates for fungal growth, development, and reproduction (Xie et al., 2018). We determined CAZymes in the SE1 genome using the dbCAN 2 meta server. A total of 313 CAZyme-encoding gene models were assigned, including 93 superfamilies, *viz*., six carbohydrate esterases, 41 glycoside hydrolases, 23 glycosyltransferases, 11 carbohydrate-binding modules, eight auxiliary activities, and four polysaccharide lyases (Table 3). There were 63 AA (auxiliary activities) genes in the SE1 genome, including 13 AA1 (multicopper oxidase), 10 AA2 (lignin-modifying peroxidase), 20 AA3 (glucose-methanol-choline oxidoreductase including cellobiose dehydrogenase, arylalcohol oxidase/glucose oxidase, alcohol oxidase, and pyranose oxidase), five AA5 (copper radical oxidase), two AA6 (1,4-benzoquinone reductase), one AA8 (cellobiose dehydrogenase), and 11 AA9 (lytic polysaccharide monooxygenase) genes.

**TABLE 3:** Carbohydrate-active enzyme annotation results.

Classification	Number	Number of superfamilies
Carbohydrate-binding module	55	11
Carbohydrate esterase	11	6
Glycoside hydrolase	140	41
Glycosyl transferase	37	23
Polysaccharide lyase	7	4
Auxiliary activities	63	8

We further investigated genes involved in the biosynthesis of secondary metabolites in *S. edulis* based on those identified in previous studies and found genes encoding fatty acid synthetases, NRPS, PKS, siderophore synthetases, and terpene synthases (Schwarzer et al., 2003; Finking and Marahiel, 2004; Sims and Schmidt, 2008; Chen et al., 2012; Lackner et al., 2012; Quin et al., 2014) that were often in contiguous gene clusters (Keller et al., 2006). Using the fungiSMASH database, we identified 39 secondary metabolite gene clusters in the SE1 genome, including four terpenes, one NRPS, one T1PKS, one siderophore, and one fatty acid gene cluster (Supplementary Table 5). Unlike primary metabolism, secondary metabolism does not participate in the growth and development of an organism but instead promotes its survival in a given environment, for example, by enhancing the defense response to pathogens. A Natural Product Domain Seeker analysis identified two C domains (C2 bacitracin_DCL and C1 cyclosporin_dual) and four KS domains (naphthopyrone_iterative, avermectin_modular, myxothiazol_modular, and epothilone_modular) in the SE1 genome.

### Phylogenetic Analysis of *S. edulis*

**FIGURE 7:**
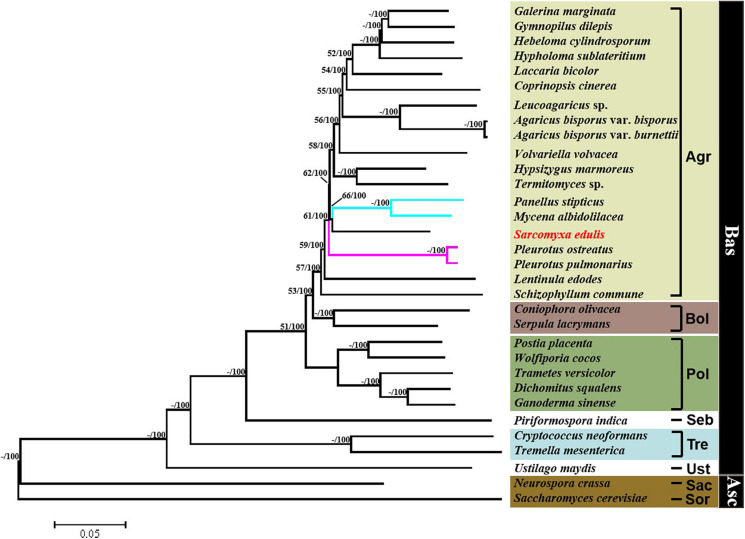
Phylogenetic tree of *Sarcomyxa edulis* and 31 other fungal species. Maximum likelihood and neighbor-joining above 50% were placed close to topological nodes and separated by “/”. The bootstrap values below 50% were labeled with “-”. Asc, Ascomycota; Bas, Basidiomycota; Sor, Sordariales; Sac, Saccharomycetales; Ust, Ustilaginales; Tre, Tremellales; Seb, Sebacinales; Pol, Polyporales; Bol, Boletales; and Aga, Agaricales.

A phylogenetic tree was constructed based on single-copy orthologous protein genes from 32 species of fungus, including 30 from the phylum Basidiomycota and two from the phylum Ascomycota serving as outgroups (Figure 7). The result showed that the *Sarcomyxa* genus formed a distinct clade to *Panellus* and *Mycena* in the *Mycenaceae* family. The topology suggested that *Sarcomyxa* was also independent of Pleurotaceae. Fungi in the Basidiomycota and Polyporales clades were clearly separated from those in the Agaricales clade, with Polyporales diverging before Agaricales. Notably, the Agaricales order followed a certain evolutionary pattern from “on log” to “on ground” growth.

## Discussion

### CAZyme Analysis

GHs, GTs, PLs, CEs, and AAs—which catalyze the modification, biosynthesis, or degradation of glycoconjugates and carbohydrates—were the main CAZymes in the SE1 genome. CMBs were non-catalytic modules that appended to the enzymes stated above. There were twice as many GH genes as there were GT genes; this may be related to the lignocellulose degradation capacity of *S. edulis* that is necessary for its survival. Polysaccharide decomposition is more important for *S. edulis* than anabolism. *S. edulis* had fewer genes related to initial lignin degradation than the average number present in Basidiomycota fungi (Supplementary Table 6; Yuan et al., 2019). The protein products of CE, GH, and PL superfamilies are involved in the breakdown of the polysaccharide portion of plant cell walls, which mainly consists of pectin, cellulose, and hemicellulose and are known as cell wall-degrading enzymes (Ospina-Giraldo et al., 2010; Yap et al., 2014). *S. edulis* had fewer candidate CAZymes than other edible fungi, but it could make better use of hardwood. Therefore, the mechanism of substance degradation of *S. edulis* needs to be further explored.

### Secondary Metabolite Analysis

Medicinal fungi have been the focus of many pharmacologic studies because of their secondary metabolites, which have antioxidant, antitumor, and antimicrobial properties. *S. edulis* has shown promising pharmacodynamic activities (Li, 1991; Inafuku et al., 2012; Kim et al., 2012; Inoue et al., 2013; Li et al., 2013, 2016; Zhang et al., 2014). Type I PKSs constitute a family of multifunctional proteins with high molecular weight and multiple catalytic domains that play an important role in the biosynthesis of reducing macrolide polyketides (Fujii et al., 2001). The chemical properties of predicted type I PKS genes in the SE1 genome have been characterized. Two C domains on contig 7—bacitracin and cyclosporine—were identified. Bacitracin is a branched cyclic peptide antibiotic active against Gram-positive bacteria and acts by binding to undecaprenyl pyrophosphate—a lipid carrier of cell wall precursors—and interfering with cell wall and peptidoglycan biosynthesis. Furthermore, bacitracin has been used for the prevention and treatment of skin and ophthalmic diseases (Ishihara et al., 2002; Hiron et al., 2011). As an immunosuppressant that is administered orally or *via* injection, cyclosporine is used to treat nephrotic syndrome (Cattran et al., 2007), psoriasis (Kumar et al., 2016), and keratoconjunctivitis sicca (dry eyes; Kasper et al., 2017). Four KS domains were also identified: naphthopyrone (KS4 on contig 12), avermectin (KS1 on contig 17), myxothiazol (KS2 on contig 17), and epothilone (KS3 on contig 17). Naphthopyrone prevents advanced glycation end product formation (Lee et al., 2006), while avermectin belongs to a family of macrocyclic lactones and functions as a biological pesticide that is highly active against a variety of nematodes. Avermectin has been widely used as an antiparasitic agent in aquaculture (Sheng et al., 2015). Myxothiazol is an antibiotic that inhibits ubiquinol. Epothilones are a class of water-soluble compounds similar to taxanes and with anticancer potential that has shown greater efficacy than paclitaxel in P-glycoprotein-expressing multidrug-resistant cell lines (Kowalski et al., 1997). At present, research on the active components of *S. edulis* are mainly focused on polysaccharides and lipids. However, study on PKS has not been reported. Therefore, the active constituents of the mushroom are still waiting to be tapped.

### Biosynthesis of Polysaccharides in *S. edulis*

The major types of bioactive compound with medicinal properties in *S. edulis* are polysaccharides such as β-D-glucan, (1→6)-β-D-glucose, and (1→3)-β-D-glucan, which have relatively high bioactivity (Ma et al., 1991). The biosynthesis of uridine diphosphate glucose—the precursor of these polysaccharides—is mediated by 1,3beta-glucan synthase and endo-1,3(4)-beta-glucanase. Two GT_48_ and 20 GH_16_ were annotated, which were present in *S. edulis* (Supplementary Table 7). The 20 GH_16_ genes were mainly predicted to be endo-1,3beta-glucanase (EC 3.2.1.39), endo-1,3(4)-beta-glucanase (EC 3.2.1.6), and endo-beta-1,3-galactanase (EC 3.2.1.181). Compared with edible and medicinal fungi, the number of GH_16_ in *S. edulis* is more than that in *Auricularia heimuer* and *W. cocos* but less than that in *Ganoderma lingzhi*. The two GT_48_ genes were predicted to be 1,3-beta-glucan synthase (EC 2.4.1.34). The same number of genes in GT_48_ was also reported in those mushrooms that contain high polysaccharide active components and have good medicinal value such as *A. heimuer*, *A. bisporus*, *G. lingzhi*, *P. ostreatus*, *V. volvacea*, and *W. cocos*.

### Phylogenetic Analysis of *S. edulis*

*Sarcomyxa edulis* is an important edible and medicinal fungus that belongs to the order Agaricus, but the taxonomy of this genus is still controversial. In order to comprehensively analyze the relationship between *S. edulis* and related species and genera, 32 fungal species from Basidiomycota and Ascomycota were used in the phylogenetic analysis. The whole genome sequences of one to two representative species from different groups of edible and medicinal fungi and several adjacent genera of *Sarcomyxa* were analyzed to show the correctness of the analysis results.

Phylogenetic analyses of the concatenated dataset using ML methods resulted in an identical and well-supported topology in all alignment strategies compared to the study of Lin et al. (2013). It can reasonably model the large stochastic processes with lower variance. Even with very short sequences, it may outperform alternative methods such as parsimony or distance methods. It also has an explicit evolutionary model for data and better accounting for branch length (Mishler, 2006). For example, Li et al. (2021) and Xu et al. (2020) used the ML algorithm in RAxML to construct a genome-based phylogenetic tree. In this study, based on the ML/NJ phylogenetic analysis of *S. edulis* and 31 other species of fungus, it was shown that the *Sarcomyxa* genus is a distinct group. This is consistent with the previous classification of *Sarcomyxa* as a clade that is independent of and emerged before the *Mycena* and *Panellus* genera based on a ML tree constructed using the D1/D2 region of *S. edulis* comb. nov. and other species (Saito et al., 2014). A study of the major clades of the Agaricales order arrived at a similar conclusion based on a maximum probability tree that combined *rpb1*, *rpb1-intron2*, *rpb2*, 18S, 25S, and 5.8S nucleotide sequences (Matheny et al., 2006). Additionally, we found that Basidiomycota, Polyporales, and Agaricales constituted distinct clades. The evolution of the Agaricales order followed a trend from “on log” to “on ground” growth.

We also observed a transition from white rot to brown rot in the Polyporales clade over the course of evolution. This is in accordance with a previous analysis of lignin-degrading peroxidases in 31 fungal genomes, which showed that the lignin degradation mechanism of white rot fungi was retained in the early evolution of Agaricomycetes, while the loss of lignin peroxidases led to the emergence of brown rot fungi exhibiting a non-ligninolytic mode of wood decay (Floudas et al., 2012; Supplementary Table 1).

The comparative analyses of secondary metabolites revealed that *S. edulis* harbors terpene and PKS biosynthesis-associated genes. The annotated whole-genome sequence of *S. edulis* can serve as a reference for identifying genes related to the synthesis of bioactive compounds with medicinal or nutritional value and the development and commercial production of superior *S. edulis* varieties.

## Data Availability Statement

The original contributions presented in the study are publicly available. This data can be found here: The genome sequence data and assembly reported in this paper are associated with NCBI BioProject: PRJNA483858 and BioSample: SAMN09748201 in GenBank. The Whole Genome Shotgun (WGS) number is INSDC: QUOL00000000.1).

## Author Contributions

FT contributed to conceptualization, methodology, software, data analysis, investigation, resources, data curation, and manuscript writing—original draft preparation. CL contributed to data validation, supervision, and project administration. CL and YL contributed to writing—review and editing. YL contributed to visualization and funding acquisition. All authors contributed to the article and approved the submitted version.

## Conflict of Interest

The authors declare that the research was conducted in the absence of any commercial or financial relationships that could be construed as a potential conflict of interest.

## Footnotes

1 http://antismash.secondarymetabolites.org

2 http://napdos.ucsd.edu

3 http://napdos.ucsd.edu/napdos_home.html

4 http://drnelson.uthsc.edu/CytochromeP450.html

## Supplementary Material

Supplementary Table 1 Summary of fungi analyzed in this study and origin of their genomes.

Supplementary Table 2 Genes predicted to be involved in non-coding RNA in *Sarcomyxa edulis*.

Supplementary Table 3 *Sarcomyxa edulis* gene annotations.

Supplementary Table 4 Putative CYP gene families of *Sarcomyxa edulis*.

Supplementary Table 5 Genes predicted to be involved in the secondary metabolism of *Sarcomyxa edulis*.

Supplementary Table 6 Distribution of the CAZyme family genes in different fungi.

Supplementary Table 7 Genes predicted to be involved in polysaccharide biosynthesis in *Sarcomyxa edulis*.
