# Supplementary material for: Genomic Analysis of Sarcomyxa edulis Reveals the Basis of Its Medicinal Properties and Evolutionary Relationships
Source: Front Microbiol. 2021 Jul 1;12:652324. doi: 10.3389/fmicb.2021.652324 (PMC8281127; doi:10.3389/fmicb.2021.652324)

Supplementary Material

# Supplementary Figures

**
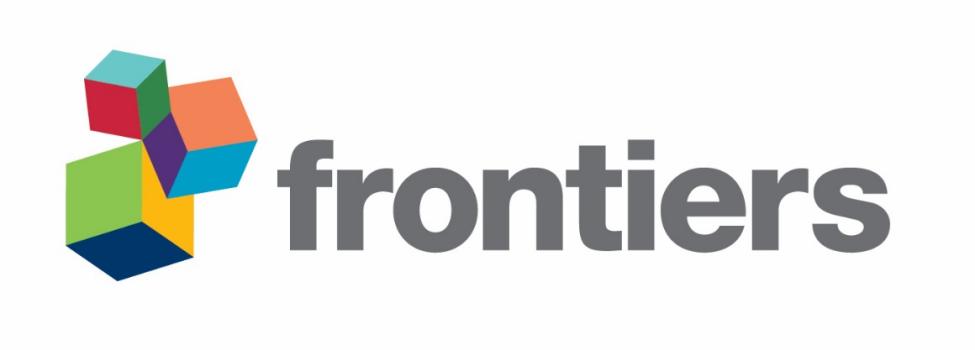
**

**Supplementary Figure 1.** Micro-characteristics of specimen 2016092521

A: Hyphae in vental trama, Bar=10 µm; B: Cystidia, Bar=20 µm; C: Cystidia with fine crystals, Bar=20 µm; D: Cystidia in ventricose, Bar=20 µm; E: Basidia and basidioles, Bar=10 µm.


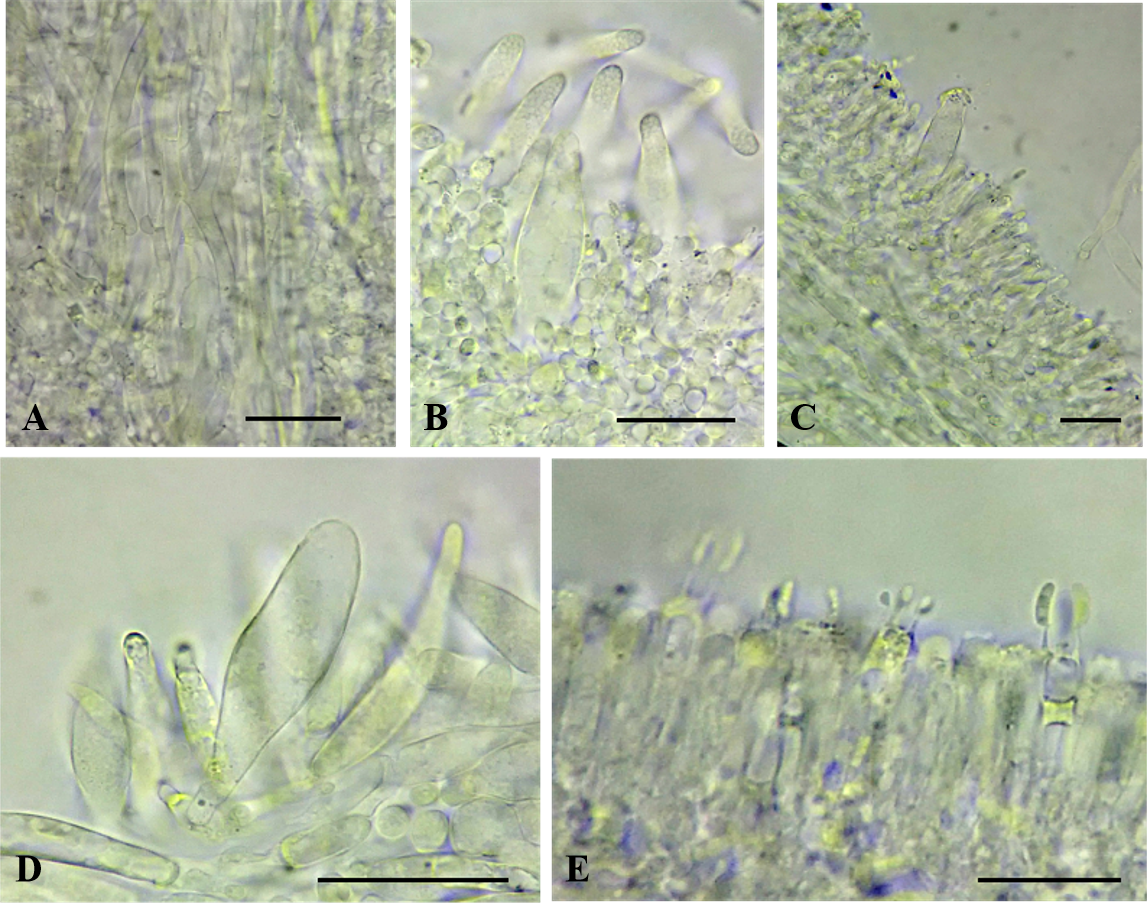


**Supplementary Figure 2.** Maximum parsimony (ML) phylogenetic tree based on ITS rRNA gene sequences, showing the position of specimen 2016092521 other related species.

The tree was rooted to *Hohenbuehelia petaloides*, *Panellus stipticus*, *Pleurotus ostreatus*, *Mycena galericulata*.


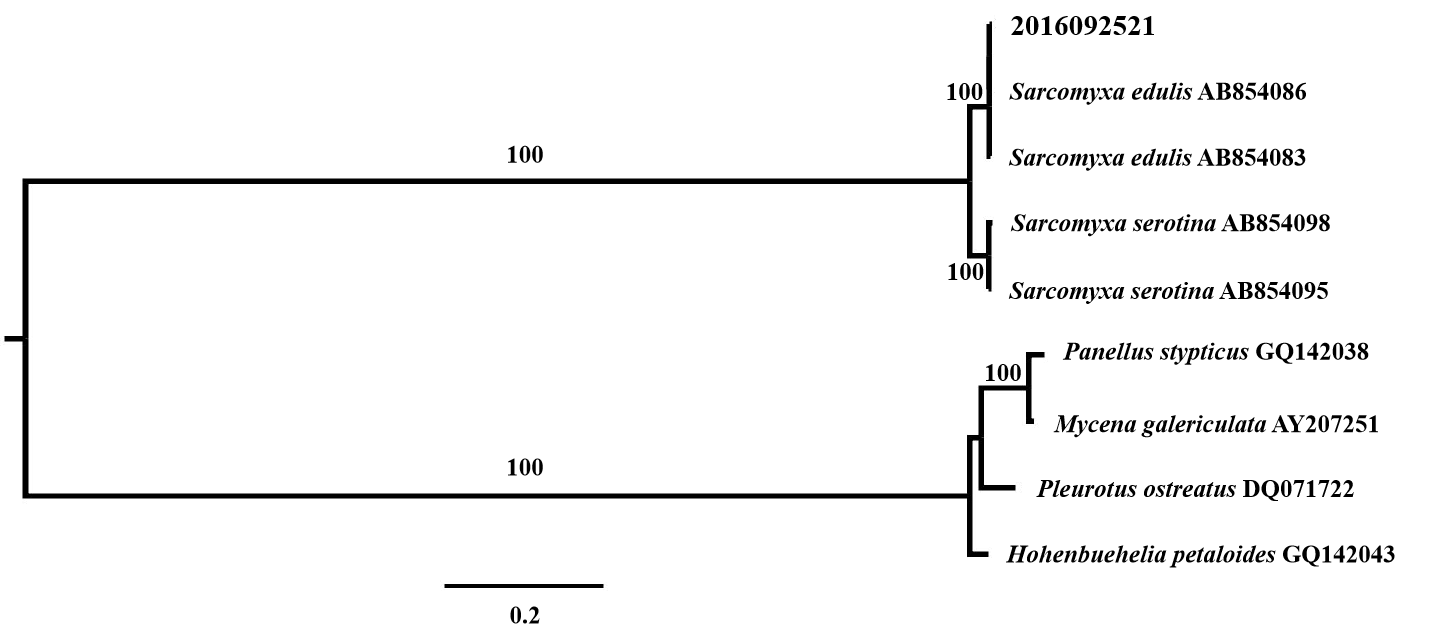

Supplement: Supplementary file 2 [file Data_Sheet_1.docx]
